# Modulations of the neuronal trafficking of tissue-type plasminogen activator (tPA) influences glutamate release

**DOI:** 10.1038/s41419-022-05543-9

**Published:** 2023-01-18

**Authors:** Alexandre Varangot, Simon Lebatard, Mathys Bellemain-Sagnard, Laurent Lebouvier, Yannick Hommet, Denis Vivien

**Affiliations:** 1 Normandie Univ, UNICAEN, INSERM U1237, Physiopathology and Imaging of Neurological Disorders (PhIND), Cyceron, Institut Blood and Brain @ Caen-Normandie (BB@C), Caen, France; 2 Department of clinical research, Caen-Normandie University Hospital, CHU Caen, France

**Keywords:** Cellular neuroscience, Protein transport, Synaptic vesicle exocytosis

## Abstract

The discovery of the neuronal expression of the serine protease tissue-type plasminogen activator (tPA) has opened new avenues of research, with important implications in the physiopathology of the central nervous system. For example, the interaction of tPA with synaptic receptors (NMDAR, LRP1, Annexin II, and EGFR) and its role in the maturation of BDNF have been reported to influence synaptic plasticity and neuronal survival. However, the mechanisms regulating the neuronal trafficking of tPA are unknown. Here, using high-resolution live cell imaging and a panel of innovative genetic approaches, we first unmasked the dynamic characteristics of the dendritic and axonal trafficking of tPA-containing vesicles under different paradigms of neuronal activation or inhibition. We then report a constitutive exocytosis of tPA- and VAMP2-positive vesicles, dramatically increased in conditions of neuronal activation, with a pattern which was mainly dendritic and thus post-synaptic. We also observed that the synaptic release of tPA led to an increase of the exocytosis of VGlut1 positive vesicles containing glutamate. Finally, we described alterations of the trafficking and exocytosis of neuronal tPA in cultured cortical neurons prepared from tau-22 transgenic mice (a preclinical model of Alzheimer’s disease (AD)). Altogether, these data provide new insights about the neuronal trafficking of tPA, contributing to a better knowledge of the tPA-dependent brain functions and dysfunctions.

## Introduction

The serine protease tissue-type plasminogen activator (tPA) is released in the blood stream from endothelial cells [1]. It is also produced by brain cells including neurons [2] and glial cells [3–6]. tPA was reported to contribute to several cerebral physiological processes [7–19] by its interaction with various receptors [20] leading mainly to modulations of the glutamatergic neurotransmission or metabolism of the extracellular matrix.

Interestingly, neuronal tPA is stored in intracellular vesicles like other proteins such as BDNF and neuropeptides [21]. In addition, tPA can be released by activated neurons [22] and recaptured from the synaptic cleft by astrocytes through a LRP-dependent mechanism [23]. In turn, astrocytes can release tPA depending on the concentration of extracellular glutamate present at the synapse [23]. When released from neurons, tPA promotes the conversion of pro-BDNF into m-BNDF at the surface of astrocytes [24]. In neurons, tPA colocalizes with synaptobrevin-2 (VAMP2) positive vesicles [25, 26], with its release found at the post-synaptic dendritic level [25]. tPA was also found associated with endocytic vesicles [25, 27]. However, the mechanisms that govern its neuronal trafficking are largely unknown.

tPA is involved in several brain disorders [20], including Alzheimer’s Disease (AD) [28–30]. Indeed, reduced levels of active tPA have been observed with ageing, a phenomenon exacerbated in AD brains (AD) [28]. Through its proteolytic activity, tPA has been reported to contribute to the degradation of A-beta [30], with its lack associated with impaired neurovascular coupling [31] and deficits on behavioral and cognitive functions in rodents [31].

Here, we provide new inputs about the neuronal trafficking of tPA, thus contributing to a better understanding of its brain functions and dysfunctions. We reveal that only the neuronal activation increases the number of VAMP2 positive and tPA-containing vesicles (V-tPA) docked at the membrane and their subsequent exocytosis, with an impact mainly observed at the post-synaptic level. When released at the synaptic cleft, tPA can thus promote release of glutamate. We also revealed that these physiological processes are dramatically altered in a model of Alzheimer’s disease, induced by Tau hyperphosphorylation.

## Results

### Neuronal activation drives the traffic of tPA-containing vesicles (V-tPA)

**Fig. 1:**
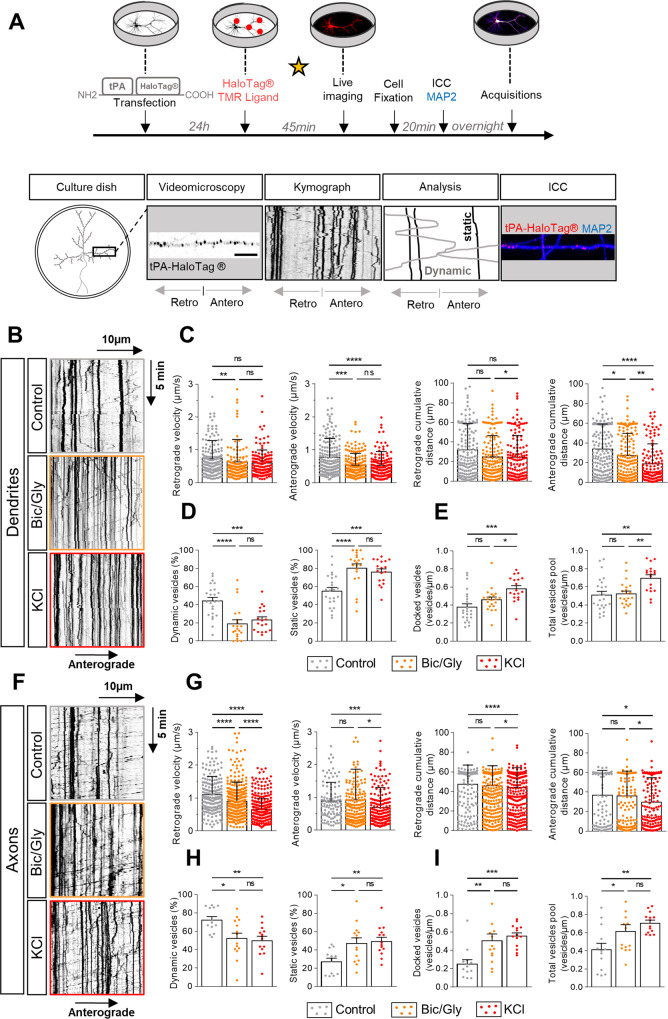
Neuronal activation influences the trafficking of tPA-containing vesicles. **A** Timeline of the experiments. The star represents the time of treatment. Cultures of cortical primary neurons and glial cells and the imaging of both dendritic and axonal tPA-HaloTag® transduced in primary cortical neurons. **B** Representative kymographs of dendritic tPA-containing vesicles after treatments: control HBBSS, bicuculline/glycine (Bic/Gly, 30 µM/200 µM respectively) and potassium chloride (KCl, 50 mM). **C** Kymographs analysis of anterograde and retrograde velocities in µm.s^−1^ and their respective cumulative distances in µm for each condition. Control *n* = 167; Bic/Gly *n* = 154; KCl *n* = 140 vesicles for retrograde trafficking and Control *n* = 177; Bic/Gly *n* = 166; KCl *n* = 138 vesicles for anterograde trafficking. **D** Percentage of dynamic or static vesicles for each dendrite. Control *n* = 24; Bic/Gly *n* = 21; KCl *n* = 18 dendrites. **E** Number of docked vesicles and total vesicles pool for each dendrite per µm. Control *n* = 24; Bic/Gly *n* = 21; KCl *n* = 18 dendrites (**C**–**E**) from 10 to 12 neurons from 2 to 5 independent cultures. **F** Representative kymographs of dendritic tPA-containing vesicles after treatments: control, Bic/Gly (30 µM/200 µM) and KCl (50 mM). **G** Kymographs analysis of anterograde and retrograde velocities in µm.s^−1^ and their respective cumulative distances in µm for each condition. Control *n* = 200; Bic/Gly *n* = 379; KCl *n* = 367 vesicles for retrograde trafficking and Control *n* = 103; Bic/Gly *n* = 185; KCl *n* = 193 vesicles for anterograde trafficking from 11 to 20 neurons from 2 to 5 independent cultures. **H** Percentage of dynamic or static vesicles for each dendrite. Control *n* = 13; Bic/Gly *n* = 14; KCl *n* = 14 axons. **I** Number of docked vesicles and the total vesicles pool for each dendrite per µm. Control *n* = 13; Bic/Gly *n* = 14; KCl *n* = 14 axons (**H**, **I**) from 13 to 14 neurons from 2 to 5 independent cultures. **C**, **G** Kruskal–Wallis’s test followed by a Dunn’s multiple comparisons test were used. Error bars = SD. **D**, **E**, **H**, **I** One-way ANOVA and a Tukey’s post hoc test were used. Error bars = SEM. **B**, **F** Kymographs scale: x = 40 µm, y = 10 min. *p < 0.05; **p < 0.01; ***p < 0.005; ****p < 0.0001, ns not significant.

We expressed a reporter tPA-HaloTag® in cultured cortical neurons (13–14 days in vitro) and revealed intracellular tPA thanks to a cell permeant fluorescent ligand (HaloTag® TMR ligand, 555Ex/585Em). Two paradigms of chemical activation were used: exposure to bicuculline/glycine (Bic/Gly, 30 µM/200 µM) or to potassium chloride (KCl, 50 mM) (Fig. 1A). Analyses of the kymographs showed a dendritic increase in the number of static V-tPA during neuronal activation, corresponding to a docking of vesicles to the plasma membrane (Fig. 1B, D, E). Bic/Gly exposure had no effect on the total pool of dendritic V-tPA while KCl exposure increased/decreased this pool (Fig. 1E). Interestingly, Bic/Gly treatment affected dendritic trafficking of mobile V-tPA by decreasing both the velocity and traveled distance of vesicles, especially in the anterograde direction (Fig. 1C). Furthermore, the intracellular increase in calcium concentration ([Ca^2+^]) caused by chemical activation increased the percentage of time spent by V-tPA on stop (Fig. S1D), shown by a tendency to reduce kymographs indices of vesicles duration state (Fig. S1A–C). A similar effect of neuronal activation by Bic/Gly or KCl exposure was also found in axons, with an increased percentage of static V-tPA (Fig. 1F, H, I) and a rise of the total pool of vesicles (Fig. 1I). Neuronal activation also reduced the velocity and the distance traveled by the vesicles in axons, but rather in the retrograde way, in particular under KCl condition (Fig. 1G). Axonal kymographs also revealed an increase in the percentage of time spent by vesicles on pause (Fig. S1G), and a reduction of kymographs indices of vesicles duration state (Fig. S1E, F). These results show that neuronal activation influences the neuronal trafficking of tPA by an increased number of static vesicles, supporting the idea that these vesicles are docked to the plasma membrane to be exocytosed “on demand”.

### Neuronal inhibition has no impact on tPA-containing vesicles trafficking, revealing a constitutive synaptic release of tPA

**Fig. 2:**
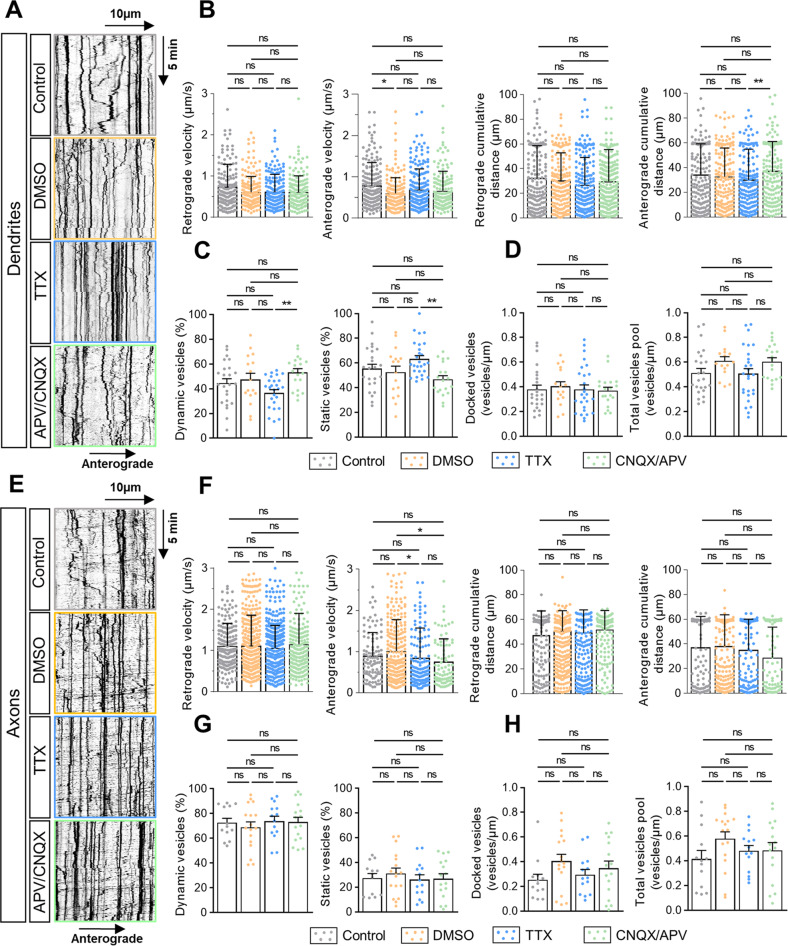
Neuronal inhibition has no impact on the trafficking of tPA-containing vesicles. **A** Representative kymographs of dendritic tPA-containing vesicles after treatments: control HBBSS, control DMSO, Tetrodotoxin (TTX, 1.5 µM) and Cyanquixaline/Acide 2-amino-5-phosphonovalérique (CNQX/APV, 50 µM/50 µM respectively). **B** Kymographs analysis of anterograde and retrograde velocities in µm.s^−1^ and their respective cumulative distances in µm for each condition. Control *n* = 167; DMSO *n* = 181; TTX *n* = 199 and CNQX/APV *n* = 155 vesicles for retrograde trafficking and Control *n* = 177; DMSO *n* = 180; TTX *n* = 212 and CNQX/APV *n* = 169 vesicles for anterograde trafficking. **C** Percentage of dynamic or static vesicles for each dendrite. Control *n* = 24; DMSO *n* = 16; TTX *n* = 27 and CNQX/APV *n* = 19. **D** Number of docked vesicles and the total vesicles pool for each dendrite per µm. Control *n* = 24; DMSO *n* = 16; TTX *n* = 27 and CNQX/APV *n* = 19 dendrites (**B**–**D**) from 11 to 14 neurons from 2 to 5 independent cultures. **E** Representative kymographs of axonal tPA-containing vesicles after treatments: control HBBSS, DMSO, TTX (1.5 µM) and CNQX/APV (50 mM/50 mM). **F** Kymographs analysis of anterograde and retrograde velocities in µm.s^−1^ and their respective cumulative distances in µm for each condition. Control *n* = 200; DMSO *n* = 549; TTX *n* = 394 and CNQX/APV *n* = 252 vesicles for retrograde trafficking and Control *n* = 103; DMSO *n* = 250; TTX *n* = 136 and CNQX/APV *n* = 81 vesicles for anterograde trafficking from 11 to 23 neurons from 2 to 5 independent cultures. **G** Percentage of dynamic or static vesicles for each axon. Control *n* = 13; DMSO *n* = 17; TTX *n* = 14 and CNQX/APV *n* = 16 axons. **H** Number of docked vesicles and the total vesicles pool for each axon per µm. Control *n* = 13; DMSO *n* = 17; TTX *n* = 14 and CNQX/APV *n* = 16 axons (**G**, **H**) from 10 to 23 neurons from 2 to 5 independent cultures. **B**, **F** Kruskal–Wallis’s test followed by a Dunn’s multiple comparisons were used. Error bars = SD. **C**, **D**, **G**, **H** one-way ANOVA and a Tukey’s post hoc test were used. Error bars = SEM. **A**, **E** Kymograph scale: x = 40 µm, y = 10 min. *p < 0.05; **p < 0.01; ***p < 0.005; ****p < 0.0001, ns not significant.

We then used two inhibitory paradigms of neuronal inhibition: tetrodotoxin (TTX, 1.5 µM) [32] or a combination of cyanquixaline (CNQX, 50 µM) and (2R)-amino-5-phosphonovaleric acid (APV, 50 µM) [33]. Both approaches had no effect on dendritic trafficking of V-tPA. We observed the same percentage of dynamic and static vesicles between control and inhibitory conditions (Fig. 2A, C). Neuronal inhibitions affected neither the number of docked vesicles nor the pool of V-tPA in dendrites (Fig. 2D). In addition, both treatments did not change the velocity and the distance traveled by the vesicles (Fig. 2B). Moreover, we observed no difference concerning kymographs indices between control and inhibitory conditions (Fig. S2A–C). Neuronal inhibition did not affect axonal trafficking, with almost the same percentage of dynamic and static vesicles with or without inhibitory treatments (Fig. 2E, G). Similarly, inhibitory treatments had no effect on the number of docked vesicles and on the pool of V-tPA present in axons (Fig. 2H). Relating to dynamic vesicles data, there was no change, excepted for the anterograde velocity, for which CNQX/APV application reduced the axonal anterograde velocity of V-tPA (Fig. 2F). Although no modification of kymographs indices was noted (Fig. S2D, E), an increase in the time spent by vesicles in the retrograde direction and a reduction in the time spent in the anterograde direction were observed with the two inhibitory treatments (Fig. S2F). Together, these data show that neuronal inhibition does not significantly affect the neuronal trafficking of V-tPA in both dendrites and axons. This unchanged tPA trafficking during inhibitory treatments are in favor of a constitutive secretion of tPA.

### Neuronal activation affects the release of tPA-containing vesicles

**Fig. 3:**
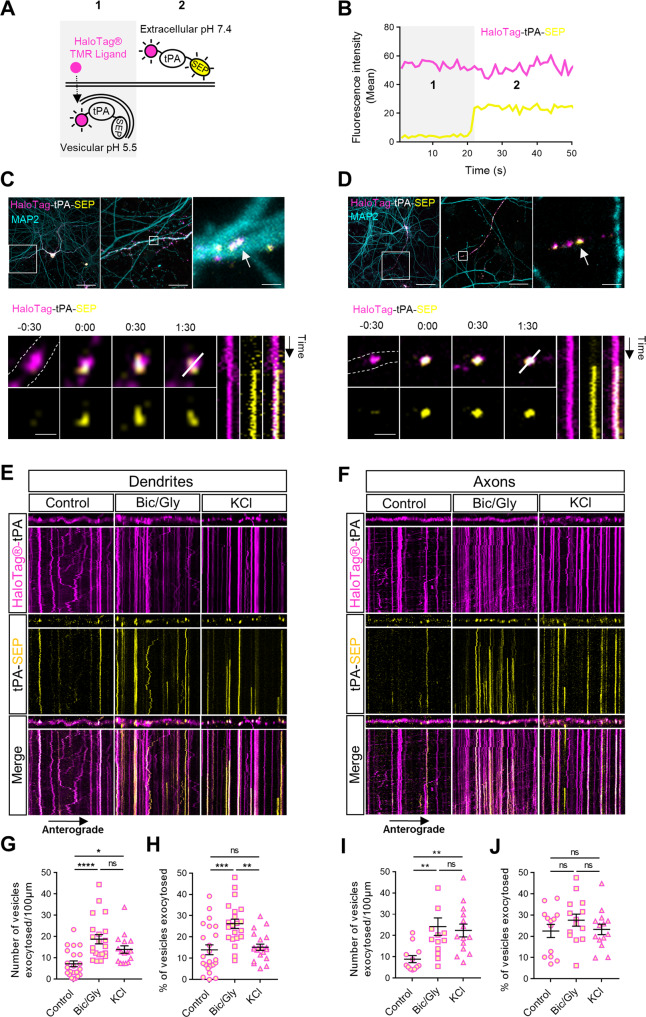
Neuronal activation increases exocytosis of tPA-containing vesicles. **A** Schematic diagrams of a dual color reporter for neuronal tPA. The halotag® allows highlighting the total pool of vesicles containing tPA, while the superecliptic pHluorin (SEP) allows observing exocytosis of vesicles, as well as the extracellular pool of tPA. **B** Graphic showing fluorescence profiles of TMR-HaloTag® and SEP before (1) and after (2) a tPA exocytosis, corresponding to the two situations in the schematic representation in (**A**). **C**, **D** Example of a dendritic tPA exocytosis in C (MAP2 positive process; in cyan) and of an axonal tPA exocytosis in (**D**) (MAP2 negative process) in basal conditions. At the top, a representative z-stack of confocal images of transfected cortical neurons (DIV13) with pCMV_HaloTag®-tPA-SEP plasmid (TMR ligand; in magenta and SEP; in yellow), a first zoom on the observed process, and a second zoom on the vesicle followed just below (white arrow). At the bottom, time-lapse images were acquired in 2 dimensions every 30 s (t = 0 was assigned of exocytosis; Time is in min:sec), nearby respective kymographs of HaloTag®-tPA-SEP exocytosis are shown. **E**, **F** Representative stacks of processes time lapse and their associated kymographs displaying HaloTag®-tPA-SEP during neuronal activation with Bic/Gly (30 µM/200 µM) and KCl (50 mM) treatments in dendrites (**E**) and in axons (**F**). All processes shown in (**E**, **F**) measures 40 µm, and all kymographs represent 40 µm horizontally and 10 min vertically. **G** Number of vesicles exocytosed per 100 µm in dendrites. Control *n* = 22, Bic/Gly *n* = 21, KCl *n* = 16 neurons. **H** Number of vesicles exocytosed (SEP positive) as the ratio of the total pool of tPA vesicles (TMR-HaloTag® positives) on dendrites. Control *n* = 22, Bic/Gly *n* = 21, KCl *n* = 16 neurons (**G**, **H**) from 2 to 5 independent cultures. **I** Number of vesicles exocytosed per 100 µm in dendrites. Control *n* = 12, Bic/Gly *n* = 14, KCl *n* = 14 neurons. **J** Number of vesicles exocytosed (SEP positive) as the ratio of the total pool of tPA vesicles (TMR-HaloTag® positives) on axons. Control *n* = 12, Bic/Gly *n* = 14, KCl *n* = 14 neurons (**G**, **H**) from 2 to 5 independent cultures. **G**, **H**, **I**, **J** one-way ANOVA and a Tukey’s post hoc tests were used. Error bars = SEM. **C**, **D** Scale bar, (top) left: 60 µm, middle: 15 µm, right: 1 µm, and (bottom) 1 µm. **E**, **F** Kymographs scale: x = 40 µm, y = 10 min. *p < 0.05; **p < 0.01; ***p < 0.005; ****p < 0.0001, ns not significant.

We then transfected neuronal cultures with a construct encoding for a dual color reporter of tPA with a HaloTag® site fused with the super-ecliptic-pHluorin (SEP). We were thus able to simultaneously observe the pool of intracellular neuronal tPA (TMR-HaloTag®-tPA) and its extracellular release (tPA-SEP) (Fig. 3A, B). We unmasked tPA exocytotic events in both dendrites (MAP2 positive) and axons (MAP2 negative) (Fig. 3C, D). Interestingly, after exocytosis, some spots of tPA remained apparent at the synapse from tens of seconds to min (Fig. 3C–F), suggesting that when released tPA stays close to the membrane. After chemical activation (with Bic/Gly or KCl), we noted an important increase in the number of docked vesicles in dendrites and in axons (Fig. 3E, F). This docking was accompanied by an increase in the number of vesicles exocytosed per µm (Fig. 3G, I) and by an increase in the number of exocytosis events on kymographs (Fig. S3A, B, F, G). Surprisingly, when considering the ratio of the number of vesicles of tPA exocytosed on the number of total V-tPA, a significant increase in tPA exocytosis was only observed at the postsynaptic level under Bic/Gly treatment (Figs. 3H, J, S3E, J). At the presynaptic level, we also observed more exocytosis of tPA (Fig. 3F, I) but because of an increased number of V-tPA in axons following activation (Fig. 3J). We also observed more exocytosis events (Fig. S3F, G) and interestingly more endocytosis events under KCl condition (S3H). These data confirm that neuronal activation leads to an increased number of docked V-tPA and subsequent exocytosis in both dendrites and axons, with an increase in the percentage of the post-synaptic release of tPA.

### Neuronal inhibition reveals a pre- and postsynaptic constitutive secretion of tPA

**Fig. 4:**
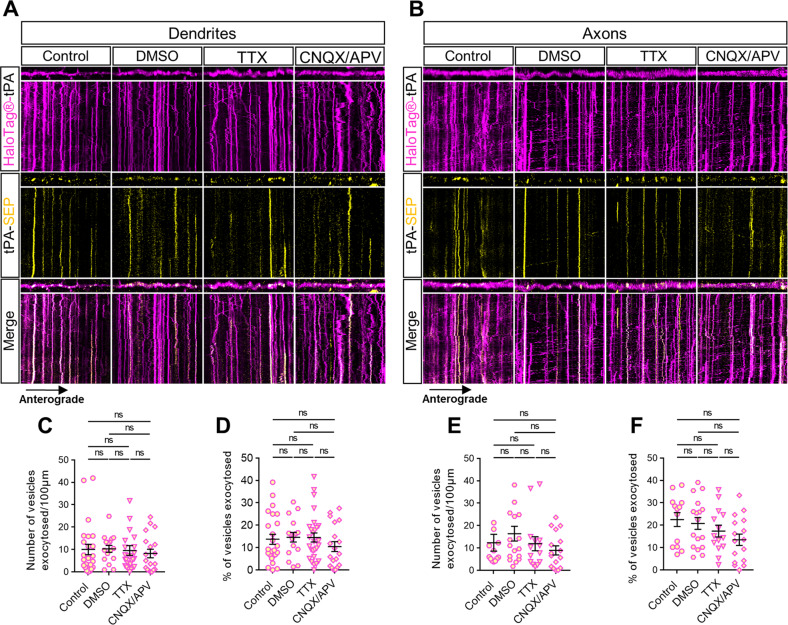
Neuronal inhibition reveals a constitutive exocytosis of tPA. **A**, **B** Representative stacks of time lapse acquisitions on neuronal processes and their associated kymographs displaying HaloTag®-tPA-SEP during neuronal inhibition with TTX (1.5 µM) and CNQX/APV (50 mM/50 mM) in dendrites (A) and in axons (B). All processes shown in A and B measure 40 µm, and all kymographs represent 40 µm horizontally and 10 min vertically. **C** Number of vesicles exocytosed per 100 µm in dendrites. Control *n* = 24, DMSO *n* = 16, TTX *n* = 27, CNQX/APV *n* = 19 neurons. **D** Number of vesicles exocytosed (SEP positive) as ratio of the total pool of tPA vesicles (TMR-HaloTag® positive) on dendrites. Control *n* = 24, DMSO *n* = 16, TTX *n* = 27, CNQX/APV *n* = 19 neurons (**C**, **D**) from 2 to 6 independent cultures. **E** Number of vesicles exocytosed per 100 µm in axons. Control *n* = 13, DMSO *n* = 17, TTX *n* = 14, CNQX/APV *n* = 16 neurons. **F** Number of vesicles exocytosed (SEP positive) as the ratio of the total pool of tPA vesicles (TMR-HaloTag® positives) on axons. Control *n* = 13, DMSO *n* = 17, TTX *n* = 14, CNQX/APV *n* = 16 neurons (**E**, **F**) from 2 to 4 independent cultures. **C**, **D**, **E**, **F** Kruskal–Wallis’s test followed by a Dunn’s multiple comparisons test were used. Error bars = SEM. **A**, **B** kymographs scale: x = 40 µm, y = 10 min. *p < 0.05; **p < 0.01; ***p < 0.005; ****p < 0.0001, ns not significant.

We then investigated whether neuronal inhibition controls exocytosis of tPA-containing vesicles (Fig. 3). After chemical inhibition (TTX 1.5 µM or CNQX 50 µM +APV 50 µM), we noted no increase in the number of docking vesicles in dendrites (Fig. 4A) and in axons (Fig. 4B) and no increase in the number of vesicles exocytosed per µm (Fig. 4C, E). We also noted no difference in the ratio of the number of vesicles of tPA exocytosed on the number of total V-tPA in both dendrites and axons (Figs. 4D, F, S3O, T). The number of exocytic and endocytic events of V-tPA were the same between control and inhibitory conditions (Fig. S3K–M, P–R). These data show that neuronal inhibition does not affect the exocytosis of tPA in both dendrites and axons. However, we observed constitutive exocytosis of tPA in both dendrites and axons with similar ranges (Fig. 4).

### VAMP2-positive vesicles and tPA-containing vesicles show similarities in their axonal anterograde trafficking suggesting their co-trafficking

**Fig. 5:**
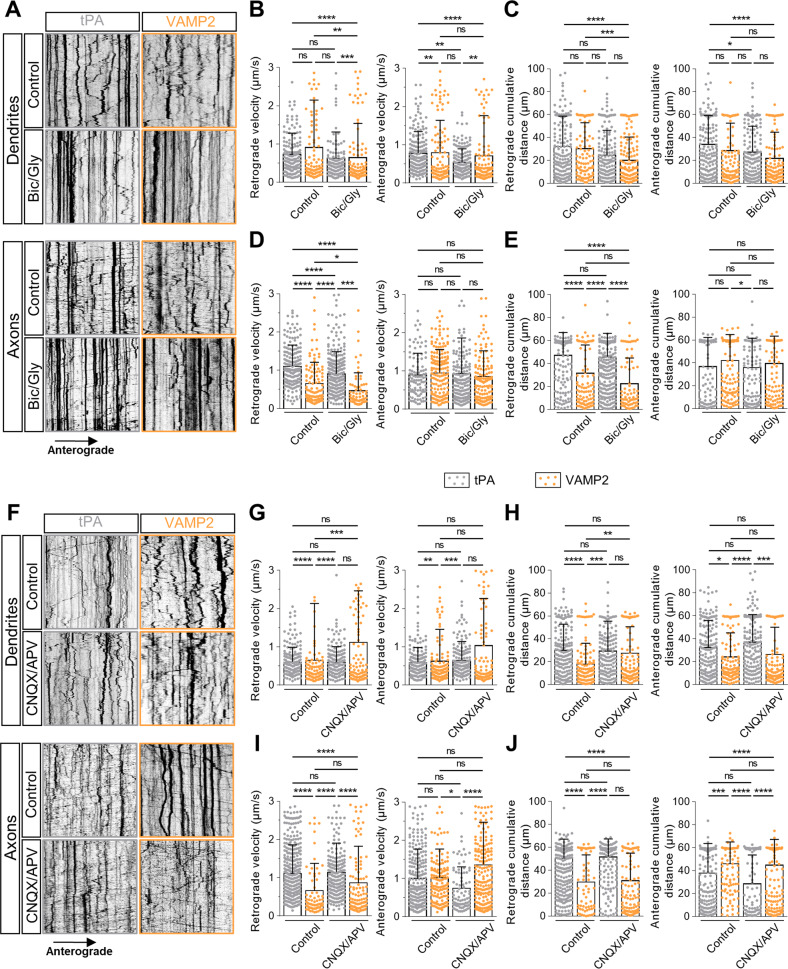
tPA-containing vesicles show similarities in their anterograde axonal trafficking under basal and activating conditions with VAMP2 positive vesicles. Representative kymographs of dendritic (upper panel) and axonal (lower panel) tPA-containing vesicles and VAMP2 positives vesicles after treatments: control HBBSS or bicuculline/glycine (Bic/Gly, 30 µM/200 µM respectively). **B** Kymographs analysis of anterograde and retrograde velocity in µm.s^−1^ and (**C**) their respective cumulative distances in dendrites in µm for each condition. Control tPA *n* = 106; Control VAMP2 *n* = 106; Bic/Gly tPA *n* = 154; Bic/Gly VAMP2 *n* = 155 vesicles for retrograde trafficking and Control tPA *n* = 177; Control VAMP2 *n* = 112; Bic/Gly tPA *n* = 166; Bic/Gly VAMP2 *n* = 144 vesicles for anterograde trafficking from 10 to 15 neurons from 3 to 5 independent cultures. **D** Kymographs analysis of anterograde and retrograde velocity in µm.s^−1^ and (**E**) their respective cumulative distances in axons in µm for each condition. Control tPA *n* = 200; Control VAMP2 *n* = 108; Bic/Gly tPA *n* = 379; Bic/Gly VAMP2 *n* = 70 vesicles for retrograde trafficking and Control tPA *n* = 104; Control VAMP2 *n* = 168; Bic/Gly tPA *n* = 185; Bic/Gly VAMP2 *n* = 136 vesicles for anterograde trafficking from 10 to 15 neurons from 3 to 6 independent cultures. **F** Representative kymographs of dendritic (high) and axonal (down) tPA-containing vesicles after treatments: control DMSO or cyanquixaline/Acide 2-amino-5-phosphonovalérique (CNQX/APV, 50 mM/50 mM respectively). **G** Kymographs analysis of anterograde and retrograde velocity in µm.s^−1^ and (**H**) their respective cumulative distances in dendrites in µm for each condition. Control tPA *n* = 181; Control VAMP2 *n* = 110; CNQX/APV tPA *n* = 155; CNQX/APV VAMP2 *n* = 118 vesicles for retrograde trafficking and Control tPA *n* = 180; Control VAMP2 *n* = 119; CNQX/APV tPA *n* = 169; CNQX/APV VAMP2 *n* = 113 vesicles for anterograde trafficking from 11 to 13 neurons from 2 to 5 independent cultures. **I** Kymographs analysis of anterograde and retrograde velocity in µm.s^−1^ and (**J**) their respective cumulative distances in dendrites in µm for each condition. Control tPA *n* = 549; Control VAMP2 *n* = 67; CNQX/APV tPA *n* = 252; CNQX/APV VAMP2 *n* = 120 vesicles for retrograde trafficking and Control tPA *n* = 250; Control VAMP2 *n* = 140; CNQX/APV tPA *n* = 81; CNQX/APV VAMP2 *n* = 219 vesicles for anterograde trafficking from 10 to 17 neurons from 3 to 5 independent cultures. **B**–**E**, **G**–**J** Kruskal–Wallis’s test followed by a Dunn’s multiple comparisons test were used. Error bars = SD. **A**, **F** kymographs scale: x = 40 µm, y = 10 min. *p < 0.05; **p < 0.01; ***p < 0.005; ****p < 0.0001, ns not significant.

tPA was reported to be present in synaptobrevin-2 (VAMP2) vesicles [25, 26] in glutamatergic neurons. We thus compared the traffic of VAMP2-positive vesicles and V-tPA in different conditions of neuronal modulation. First, we analyzed retrograde and anterograde trajectories of tPA and VAMP2 in dendrites and in axons following application of Bic/Gly (30 µM/200 µM). Bic/Gly increased the docking of these vesicles (Fig. 5A). Interestingly, we also observed that VAMP2- and V-tPA displayed the same average anterograde velocity (Fig. 5A, D) and the same average cumulative distance traveled (Fig. 5A, E) in control and in activating conditions. Nevertheless, when we focused on the times spent by axonal vesicles in both anterograde and retrograde directions, V-tPA appeared predominantly retrograde while VAMP2-containing vesicles were largely anterograde (Fig. 5A, S5B). Neuronal activation lead to an overall decrease in the speeds and distances traveled by the two types of vesicles (Figs. 5B–E, S5A). During neuronal inhibition (CNQX/APV (50 µM/50 µM)), tPA and VAMP2-containing vesicles differed in their characteristics. Indeed, although neuronal inhibition failed to influence the trafficking of V-tPA (Figs. 2, S2), an increase in the average retrograde velocity (Fig. 5F, G) and of the average retrograde cumulative distance (Fig. 5F, H) was observed for dendritic VAMP2 positive vesicles, associated with a reduction in the percentage of vesicles pausing time (Fig. S5C). Unlike activation, inhibition also revealed a different pattern of axonal anterograde transport between tPA and VAMP2. We observed a higher average anterograde velocity (Fig. 5F, I) and a higher average distance traveled (Fig. 5F, J) for VAMP2 positive vesicles compared to V-tPA. As observed under neuronal activation, axonal VAMP2 positive vesicles spent most of their time in the anterograde way while axonal V-tPA spent most of their time in the retrograde way (Fig. S5D). During inhibition the two types of vesicles spent the same pausing time (Fig. S5D). All these data suggest that VAMP2 and tPA present a co-trafficking in axonal anterograde way in both neuronal resting state or activating state. When neurons are inhibited, traffic parameters indicated that VAMP2 vesicles display a pattern, particularly in dendrites, at the opposite of V-tPA.

### Exogenous tPA promotes the release of VGlut1 positive vesicles from cultured cortical neurons

**Fig. 6:**
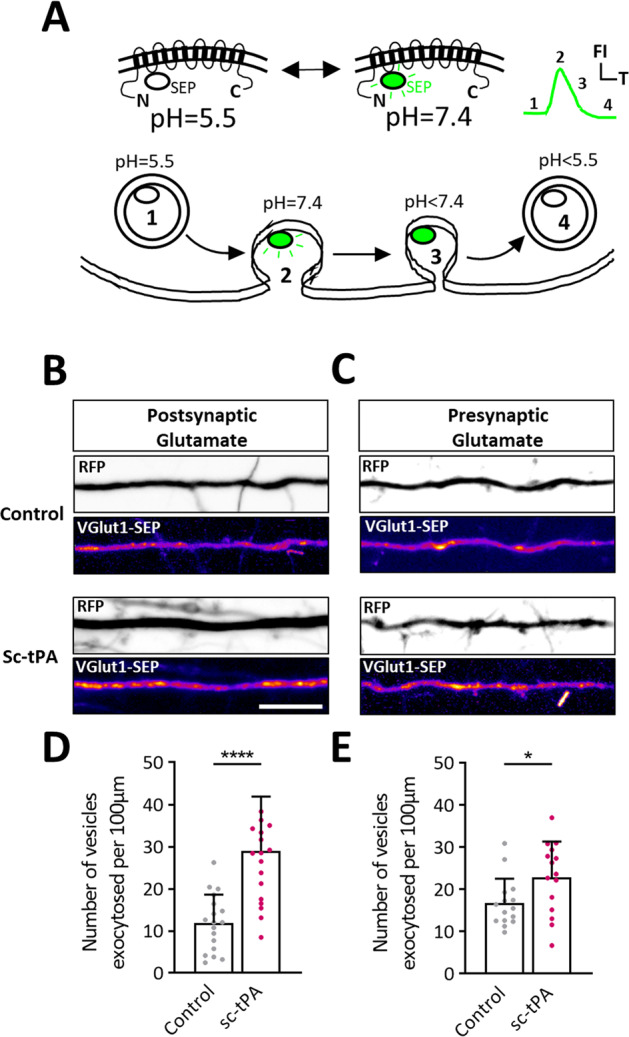
tPA increases the pre and postsynaptic release of VGlut1 vesicles. **A** Schematic representation and function of VGlut1-SEP to measure the exocytosis of glutamate-containing vesicles. **B**, **C** Representative stacks of processes time lapse displaying a RFP (black and white) and Vglut1-SEP (pseudo-color) signal during control HBBSS or tPA (300 nM) treatment. **D** Quantifications of the average number of VGlut1-SEP positives spots per 100 µm in dendrites in control HBBSS condition or after addition of exogenous tPA (300 nM). Control *n* = 18, tPA *n* = 19 dendrites from 10 to 12 neurons from 3 to 4 independent cultures. **E** Quantifications of the average number of VGlut1-SEP positives spots per 100 µm in axons in control HBBSS condition or after addition of exogenous tPA (300 nM). Control *n* = 15, tPA *n* = 15 dendrites from 15 neurons from 4 independent cultures. Statistical tests: unpaired t test, *p < 0.05; ****p < 0.0001. Error bars indicate SD. Scale bar = 10 µm.

Here, we wanted to know if tPA influences the release of glutamate by cortical neurons. We used the vesicular glutamate transporter 1 (VGlut1) coupled with a pHluorin to follow in real time the exocytosis of glutamate (Fig. 6A). Using live spinning disk imaging, we were capable to reveal, in high timing resolution, the fusion events of VGlut1-SEP positive vesicles in both dendrites and axons with or without tPA treatment (Fig. 6B, C). tPA treatment (300 nM) significantly increased the average number of VGlut1-SEP positive vesicles presents at the surface of dendrites (Fig. 6D) and axons (Fig. 6E). These results showed that tPA can increase the release of synaptic glutamate.

### Alteration of the axonal trafficking of tPA-containing vesicles in cultured neurons from the THY-Tau22 mouse model of AD

**Fig. 7:**
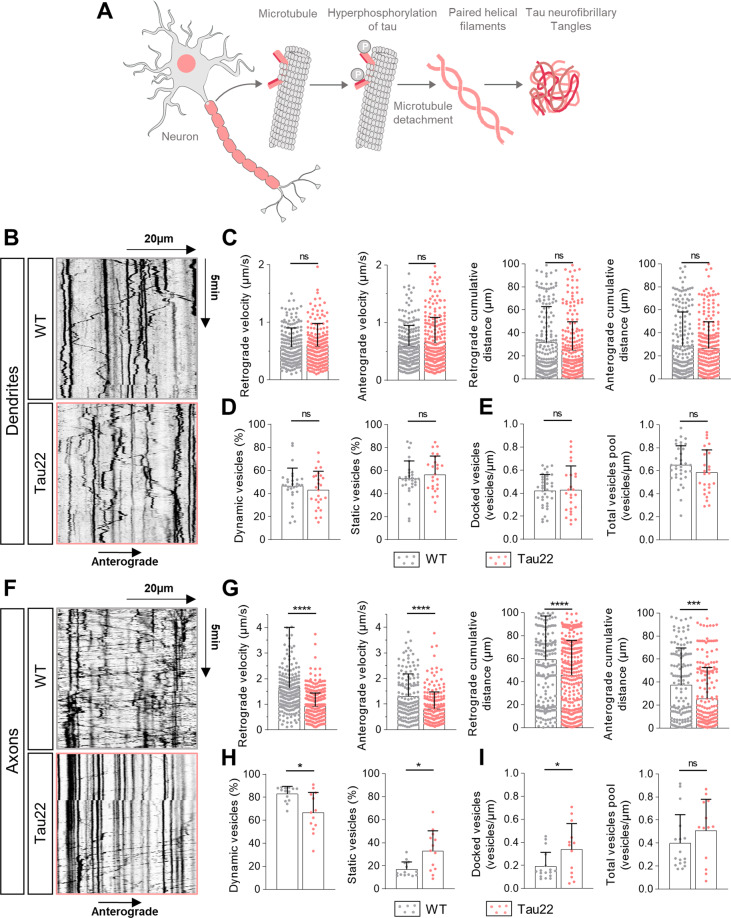
The trafficking of the axonal tPA-containing vesicles is altered in cultured neurons prepared from the Tau22 mouse model of tauopathy. **A** Diagram showing the formation of neurofibrillary tangles (NFTs) by the hyperphosphorylation of tau protein in the mouse model Tau22 of tauopathy (inspired from Abcam). **B** Representative kymographs of dendritic tPA-containing vesicles in WT neurons and Tau22 neurons. **C** Kymographs analysis of anterograde and retrograde velocity in µm.s^−1^ and their respective cumulative distance in µm for each condition. WT *n* = 196; Tau22 *n* = 241 vesicles for retrograde trafficking and WT *n* = 210; Tau22 *n* = 241 vesicles for anterograde trafficking. **D** Percentage of dynamic or static vesicles for each dendrite. WT *n* = 18; Tau22 *n* = 19. **E** Number of docked vesicles and the total vesicles pool for each dendrite per µm. WT *n* = 18; Tau22 *n* = 19 (**C**–**E**) from 15 to 16 neurons from 3 to 4 independent cultures. **F** Representative kymographs of axonal tPA-containing vesicles in WT neurons and Tau22 neurons. **G** Kymographs analysis of anterograde and retrograde velocity in µm.s^−1^ and their respective cumulative distances in µm for each condition. WT *n* = 252; Tau22 *n* = 377 vesicles for retrograde trafficking and WT *n* = 144; Tau22 *n* = 201 vesicles for anterograde trafficking from 13 to 14 neurons from 3 to 4 independent cultures. **H** Percentage of dynamic or static vesicles for each axon. WT *n* = 13; Tau22 *n* = 14. **I** Number of docked vesicles and the total vesicles pool for each axon per µm. WT *n* = 13; Tau22 *n* = 14 (**H**, **I**) from 13 to 14 neurons from 3 to 4 independent cultures. **C**, **D**, **E**, **G**, **H** Samples were drawn from at least three independent experiments. Statistical tests: two-tailed Mann–Whitney test, *p < 0.05; ***p < 0.005; ****p < 0.0001, ns not significant. Kymograph scales: x = 40 µm, y = 10 min.

We investigated the trafficking of neuronal tPA in cultured neurons prepared from the THY-Tau22 (Tau22) mouse model of AD, previously characterized by an hyperphosphorylation of Tau, that ends up in unhooking of microtubules, Tau aggregates, paired helical filaments and neurofibrillary tangles (NFTs) [34] (Fig. 7A). At the dendritic level, we observed the same percentage of dynamic and static V-tPA between wild-type (WT) and Tau22 cultured cortical neurons (Fig. 7B, D). Similarly, tauopathy affected neither the number of docked vesicles nor the total pool of V-tPA in dendrites (Fig. 7B, E). By examining the dynamic of these vesicles, the tauopathy changed neither the velocity nor the distance traveled by the vesicles (Fig. 7C). Moreover, there was no difference concerning the other kymograph settings (persistence, % of time spend by vesicles in each direction) between dendrites of WT and Tau22 cortical neurons (Fig. S5A, B, D). However, microtubule destabilization induced by Tau hyperphosphorylation in corresponding axons led to an impaired trafficking of V-tPA, with an increased percentage in static vesicles (Fig. 7F, H) and in the number of docked vesicles for a same amount of V-tPA (Fig. 7I). Tau22 neurons presented a drastic decrease in both anterograde and retrograde velocities of V-tPA and of the cumulative distance traveled by these vesicles (Fig. 7G). We also revealed the same modifications of kymographs indices (Fig. S5E–G), and an increase in the time spent by dynamic V-tPA in pause in Tau22 axons (Fig. S5H). Taken together, these data suggest that hyperphosphorylated tau leads to an important alteration of the axonal trafficking of V- tPA.

### Alteration of axonal V-tPA trafficking in Tau22 neurons promotes the exocytosis of axonal tPA

**Fig. 8:**
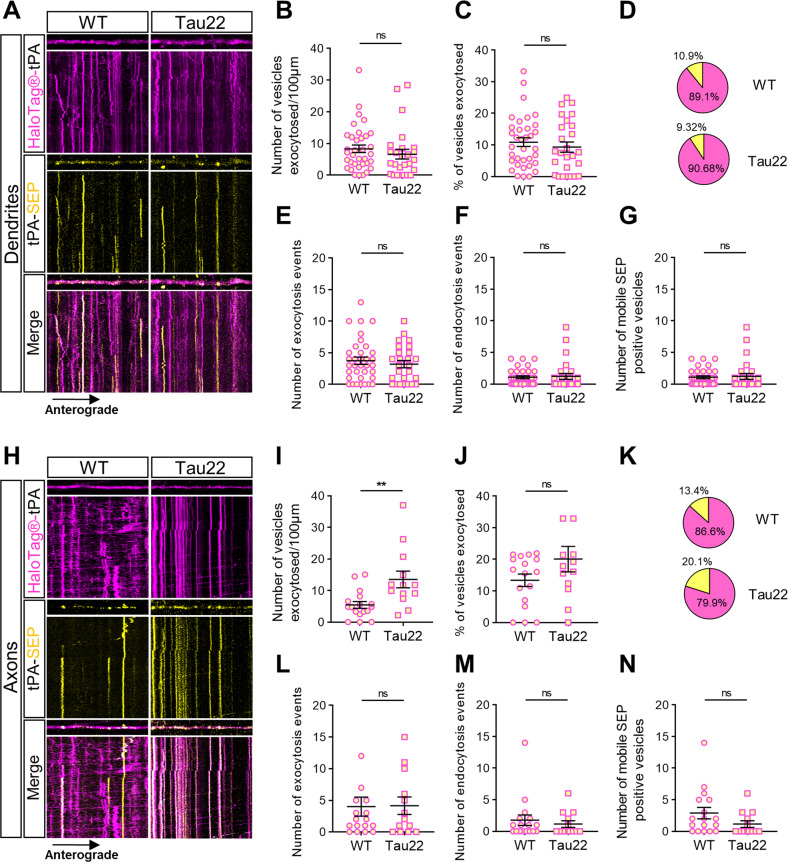
Tauopathy increases exocytosis of axonal tPA. **A**, **H** Representative stacks of processes time lapse and their associated kymographs displaying HaloTag®-tPA-SEP in WT and Tau22 neurons in dendrites (**A**) and in axons (**H**). All processes shown in (**A**, **H**) measure 40 µm, and all kymographs represent 40 µm horizontally and 10 min vertically. **B** Number of vesicles exocytosed per 100 µm in dendrites. **C** Number of vesicles exocytosed (SEP positive) as the ratio of the total pool of tPA vesicles (TMR-HaloTag® positives) on dendrites. **D** Pie charts representing the percentage of exocytosed HaloTag-tPA-SEP vesicles (SEP positive, in yellow) and intracellular HaloTag-tPA-SEP vesicles (SEP negative, in magenta) in dendrites. **E**, **F** Number of tPA-SEP exocytosis (**E**) and endocytosis (**F**) events observed in basal conditions. **G** Number of dendritic motile tPA-SEP positives vesicles. (**B**–**G**) WT *n* = 20 and Tau22 *n* = 19 (**C**, **D**) from 3 independent cultures. **I** Number of vesicles exocytosed per 100 µm in axons. **J** Number of vesicles exocytosed (SEP positive) as ratio of the total pool of tPA vesicles (TMR-HaloTag® positives) on axons. **K** Pie charts representing the percentage of exocytosed HaloTag-tPA-SEP vesicles (SEP positive, in yellow) and intracellular HaloTag-tPA-SEP vesicles (SEP negative, in magenta) in axons. **L**, **M** Number of tPA-SEP exocytosis (**L**) and endocytosis (**M**) events observed in axons in basal conditions. **N** Number of axonal motile tPA-SEP positives vesicles. **I**–**N** WT *n* = 14 and Tau22 *n* = 13 (**C**, **D**) from 3 independent cultures. **B**–**G**, **I**–**K** Samples were drawn from at least three independent experiments. Statistical tests: two-tailed Mann–Whitney test, **p < 0.01, ns not significant. Kymograph scales: x = 40 µm, y = 10 min.

We then examined whether this Tau22 mutation causes, in addition to an alteration of the axonal trafficking, a dysregulation of V-tPA exocytosis. We observed no significant difference in the number of exocytosed V-tPA per µm (Fig. 8A, B), and no difference in the percentage of exocytosed V-tPA relative to the total pool of V-tPA in dendrites of Tau22 neurons when compared to WT neurons (Fig. 8C, D). The number of exocytosis and endocytosis events and the number of motile SEP positive vesicles were also identical (Fig. 8E, F) in WT and Tau22 neurons. In axons, we observed an increase in the number of exocytosed V-tPA per µm in Tau22 neurons (Fig. 8H, I) but no difference in the percentage of exocytosed V-tPA relative to the total V-tPA pool (Fig. 8J, K) showing an accumulation of these vesicles. These results indicate that we revealed more V-tPA exocytosis in Tau22 axons because of a larger pool of total V-tPA compared to WT axons. We also revealed no difference in the number of endocytosis and exocytosis events (Fig. 8L, M) as well as in the number of mobile exocytosed V-tPA between Tau22 and WT neurons (SEP positive) (Fig. 8N). These data show that the THY-Tau22 model provokes mainly axonal disorders including an increase of the docking of V-tPA and of a subsequent increase in their exocytosis.

## Discussion

In this study, we investigated the trafficking and secretion of tPA in cortical neurons under physiological and pathological situations. Neuronal activation affected dendritic and axonal tPA traffickings. We noticed that Bic/Gly treatment influences the bidirectional tPA trafficking in dendrites while at the axonal level it is the KCl treatment. These observations are in agreement with the expected actions of these treatments. Although biccucculin is expected to inhibit GABA_A_ receptors of inhibitory interneurons, glycine is expected to enrich the postsynaptic currents by activating NMDARs [35, 36]. Thus, the combination of biccuculin and glycine should lead to a postsynaptic depolarization. The addition of KCl is expected to increase the number of action potentials at the presynaptic terminals, leading to a post-synaptic depolarization [37, 38]. This behavior of V-tPA trafficking is correlated to an increase of exocytosis in both dendrites and axons (Figs. 3, S3). We agree with the literature showing that depolarization induced by bicuculline or by a high concentration in K+ increased the neuronal release of tPA [39–41]. We also observed that the exocytosis of tPA caused by chemical activations reveal different release mechanisms at the dendritic and axonal levels. At the dendrite, an increase in exocytosis is observed with the same number of vesicles like control condition which mobilizes the vesicles pool, while in axons, activation increases the number of vesicles and docking vesicles present on processes (Fig. 3). Neuronal inhibition has no effect on V-tPA trafficking and exocytosis (Figs. 2, S2, S3, 4).

The axonal anterograde V-tPA show similarities with VAMP2 positive vesicles in resting and activating state of neurons suggesting a co-trafficking in the same vesicles (Figs. 5, S4). tPA has been identified in dense core vesicles (DCVs) [42] and in a subset of DCVs-containing VAMP2 [26, 43]. We found that tPA and v-SNARE VAMP2 had the same axonal anterograde vesicle velocity and the same vesicle distance traveled in control and in activating conditions (Fig. 5). In opposite to V-tPA, VAMP2 positive vesicles responded well to inhibitory treatments. Interestingly, despite common axonal anterograde motor parameters, the majority of axonal VAMP2 positive vesicles are anterograde [44] and V-tPA retrograde (Figs. 1, 2, 5; Lenoir et al. [25]). This is explained by the function of these two proteins: VAMP2 is predominantly present in the secretion and fusion compartment, while tPA is found in all compartments in dendrites and axons. tPA is described to be co-localized with endosomal family markers protein Rab, notably tPA is found in early endosomes positive for Rab5, late endosomes (Rab7) and in recycling endosomes (Rab 11) in both axons and dendrites [25]. The abundant retrograde trajectories representative of axonal kymographs of tPA resemble closely of signaling endosomes trajectories with the same average speed [45]. Our data also suggest that during a neuronal inhibition, the potential axonal constitutive secretion of tPA passes through organelles other than VAMP2 positive DCVs.

We also observed that exogenous tPA can increase the exocytosis of VGlut1 positive vesicles containing glutamate (Fig. 6), consistent with its capacity to modulate glutamatergic transmission [46]. In agreement with the literature, we observed VGlut1 positives vesicles in the postsynaptic compartment [47, 48]. Addition of tPA led to an increase in the fusion of VGlut1-SEP positive vesicles at both the dendritic and axonal surfaces. The existence of presynaptic NMDARs is also reported [49], explaining our presynaptic observations. tPA may interact with both pre- and post-synaptic NMDARs to increase glutamate release in dendrites and axons.

All of our present experiments were performed on mixed cell cultures containing astrocytes. Astrocytes are able to recapture and recycle synaptic tPA, depending on extracellular glutamate levels [23]. Moreover, astrocytes are also described to concentrate plasminogen on their surface, which can be activated by tPA released from astrocytes and neurons [24]. Plasmin then generated is able in turn to activate other molecules including growth factors like pro-BDNF in mature BDNF [50], known to promote neuronal and synaptic growth, to maintain LTP and to promote the release of glutamate [51–54].

We finally studied the trafficking of tPA in THY-Tau22 cortical neurons presenting an increase in the hyperphosphorylation of tau. We observed that aberrant Tau phosphorylation affects the axonal trafficking of tPA, with a large reduction of V-tPA velocity and distance traveled (Fig. 7). Moreover, trajectories of axonal V-tPA showed an increase of pausing time (Fig. S5) with an increase in the number of V-tPA docked in THY-Tau22 axons (Fig. 6). These results correlated with an increased exocytosis of tPA in THY-Tau22 neurons (Fig. 8). These observations could explain the similarities found in the behavior of tPA KO and THY-Tau22 mice such as deficits in spatial memory [55, 56]. We found no alteration of the trafficking and exocytosis of dendritic tPA, probably due to the absence of NFTs formation at this stage. It was described in THY-Tau22 mice that NFTs are formed around 6 months [34]. It is very likely that the 14 days of in vitro life of our cultured neurons are not enough for NFTs formation, but sufficient to observe heavy deficits in axonal trafficking. Our data are in agreement with the recent literature in other AD models reporting a decrease in the vesicular speeds in THY-Tau22 axons [57].

Altogether, we provide here a robust description of the trafficking of tPA in cortical neurons under different neuronal activities. We thus described both presynaptic and postsynaptic behaviors of V-tPA and showed that they are altered in a mouse model of AD.

## Material and methods

### Animals

THY-Tau22 mice are characterized by the overexpression of human 4-repeat Tau mutated at sites G272V and P301S under the control of Thy1.2 promoter [34]. All Tg mice used in the present study were heterozygous. Non-Tg littermates were used as wild-type (WT) controls for all experiments. The chosen line (THY-Tau22) is fertile with normal frequency and size of litters and stably transmits the transgene to its offspring.

### Reagents

Recombinant human tPA (Actilyse®) was purchased from Boehringer Ingelheim (Ingelheim am Rhein, Germany). Fetal bovine serum, horse serum, lipofectamine R 2000 reagent, B27 supplement, glutamine, laminin, neurobasal medium, and penicillin/streptomycin were purchased from ThermoFisher (Waltham, Massachusetts, USA). Dulbecco’s modified Eagle’s medium (DMEM), poly-D-lysine, phosphate-buffered saline (PBS), Glycine (Gly), paraformaldehyde, albumin from bovine serum, ammonium chloride (NH4Cl), potassium chloride (KCl), and rabbit polyclonal antibody were purchased from Sigma-Aldrich (St Louis, MO, USA). Tetrodotoxin citrate (TTX), cyanquixaline (CNQX), (2R)-amino-5-phosphonovaleric acid (APV) and bicuculline methiodide (Bic) were purchased from Tocris (Bristol, UK). HaloTag® TMR ligand was purchased from Promega (Madison, Wisconsin, USA). The following primary antibodies were used for immunocytochemistry: mouse monoclonal anti-HaloTag® (dilution 1:1,000; Promega; G9211) rabbit anti-tPA polyclonal antibody (dilution 1:1 500; generous gift from R. Lijnen, Leuven), chicken anti-Microtubule-associated protein 2 (MAP2) polyclonal antibody (dilution 1:8 000; Abcam, Cambridge, UK; ab5392). Secondary fluorescent antibodies (Alexa647; dilution 1:800) were purchased from Jackson Immunoresearch (Bar Harbor, ME, USA).

### Plasmid constructs

The cDNA encoding for amino acids 1 to 32 of the peptide signal of human tPA was amplified from the full-length human tPA cDNA. The corresponding Polymerase Chain Reaction (PCR) product was subcloned into the eukaryotic expression plasmid pCDNA5.1 between NheI and BamHI. Then, the full-length coding sequence for mature human tPA was PCR amplified and subcloned downstream of the “peptide signal−6xHis” between BamHI and XhoI restriction sites to generate a cDNA encoding for the mature 6xHis human tPA (tPAwt). The cDNA of HaloTag® without the Tobacco Etch Virus protease (TEV) cleavage site was PCR amplified from the plasmid pFC14A (Promega, France) and subcloned into pCDNA5.1-tPA between the end of the sequence encoding for tPA and the STOP codon at the end of the Halo-Tag®, then fused in BamHI between the His-tag and the beginning of the cDNA for tPA (HD-Fusion technologies from Clontech, USA). For synapsin promote-driven constructs, we replaced the CMV promotor of the plasmid pCI by the cDNA of the synapsin promotor subcloned between BglII and NheI. For pCMV_HaloTag®-tPA-SEP, the cDNA encoding for the pH sensitive GFP (pHluorin) was subcloned by fusion into pCMV_HaloTag®-tPA between the end of the sequence of tPA and the STOP codon. The constructs of pCMV_GFP_VAMP2, and pCAG_RFP were provided by Thierry Galli (Paris, France). The constructs of pCMV_SEP_VAMP2 and pCMV_SEP_VGlut1 were provided by Frédéric Saudou (Grenoble, France) and Timothy Ryan (New York, USA) respectively. pCMV_GFP (pAcGFP1-N1 Vector, Cat#632469) was purchased from Takara Bio (Kusatsu, Japan). All the constructs were amplified in Escherichia coli DH5a cells and purified by a Nucleobond endotoxin-free plasmid DNA PC 2000 kit (Macherey-Nagel) according to the manufacturer’s instructions.

### Cell cultures

Cortical astrocyte cell cultures were prepared from 1 to 3 days postnatal SWISS mice. Cerebral cortices were dissected and dissociated in DMEM. Then, cells were plated in DMEM supplemented with 10% fetal bovine serum, 10% horse serum and 2 mM glutamine on poly-D-lysine (0.1 mg/ml) and laminin (0.02 mg/ml)-coated T75 Flasks. The medium was changed two times weekly until the cell reach confluence, after cells were maintained in DMEM supplemented with 5% fetal bovine serum, 5% horse serum and 2 mM glutamine. To maintain neuronal cells without serum (glio-conditioned medium), astrocytes cultures are incubated over night with Neurobasal Medium supplemented with 0.4 mM glutamine, 2% B27 supplement 50X and penicillin streptomycin (10,000 IU/ml; 10,000 UG/ml).

Primary cultures of cortical neurons were prepared from fetal SWISS mice (embryonic day 14) as previously described in Buisson et al. [3]. Cortices were dissected and dissociated in DMEM and plated (250,000 cell/mL) on glass bottom microwell dishes (MatTek Corporation, P35G-1.5-14-C, Ashland, MA, USA) earlier coated with polyD-lysine (0.1 mg/ml) and laminin (0.02 mg/ml). Cells were cultured in Neurobasal Medium supplemented with 0.4 mM of glutamine, 2% B27 supplement 50X, 10% horse serum and penicillin/streptomycin (10,000 IU/ml; 10,000 UG/ml). After 1 h, media were replaced by glio-conditioned medium obtained from primary cultures of astrocytes (see above). Cultures were maintained at 37 °C in a humidified 5% CO2 atmosphere. One third of medium was changed one time weekly by fresh glio-conditioned medium.

### Neuronal Transfection

Transfections were performed at Day In Vitro (DIV) 12 or DIV 13. Neuronal cultures were washed with HEPES and Bicarbonate Buffered Salt Solution (HBBSS; NaCl: 116 mM, KCl: 5.4 mM, CaCl_2_: 1.8 mM, MgSO_4_: 0.8 mM, HEPES: 12 mM, NaH_2_PO_4_: 0.34 mM, D-glucose: 5.5 mM, NaHCO_3_: 25 mM and Glycine: 10 µM) prior to an 8 h incubation in the presence of the mentioned cDNAs and lipofectamine R 2000-containing HBBSS. Regular media as described above (cell cultures section) then replaced HBBSS. The transfection efficiency was 10–20%.

### tPA-HaloTag® Detection with HaloTag® TMR Ligand

After 24–48 h, neuronal cultures were washed 3 times with HBBSS, HaloTag® TMR ligand was added during 15 min and neuronal cultures were washed 3 times again with HBBSS (to remove unbound ligand) and 30 min later a last wash was performed 3 times with HBBSS (to remove astrocytic release of captured unbound ligand).

### Immunocytochemistry

Neuronal cultures were washed with HBBSS, fixed in paraformaldehyde 4% for 20 min at room temperature, washed in PBS (0.1 M) and blocked 1 h in PBS containing 0.3% Triton X100 and albumin (4%). The primary and secondary fluorescent antibodies were used (see reagent section) and incubated on neuron cultures in PBS containing 0.15% Triton X100. Between these incubations, cultures were rinsed 3 times and washed 2 times during 10 min in PBS (0.1 M). After confocal laser-scanning microscopy was performed (see the next section).

### Microscopy

#### Laser-Scanning Confocal Microscopy

Laser-scanning confocal microscopy (LSCM) was performed using an inverted Leica SP5 confocal microscope and a Leica TCS SP8 Confocal/STED microscope (Leica Microsystems SAS; Leica, Wetzlar, Germany) equipped with an Argon Gas laser and a X40 NA = 1.3 oil immersion objective. Cultures were scanned at room temperature with 488-, 561, 633-nm, laser lines to detect the GFP, SEP, HaloTag® TMR ligand and Alexa647, respectively and high-resolution images (2048 × 2048, 12 bits) of optical sections (z stack, step: 0.45 µm) were captured using sequential line average (mean of three) scanning. Colocalizations of two or three fluorophores were qualitatively assessed in the x, y, and z planes of each optical section. Maximal projection images of confocal z series (stacks) were generated where indicated in the figure legends. Minimal adjustments to image contrast and intensity were made in ImageJ software using the levels or contrast/brightness functions.

#### Live imaging

Live imaging experiments were achieved using 3 different microscopes. Protein trafficking and fusion acquisitions were performed with a Leica SP5 confocal microscope and a Leica TCS SP8 Confocal/STED microscope (Leica Microsystems SAS; Leica, Wetzlar, Germany) using a ×40 oil immersion objective. All time-lapse images were collected with the same parameters on these two microscopes at a 512 × 512–pixel resolution (8 bit). A short interval (1.29 s) for 465 images in a sequential mode was used to maximize vesicles resolution trajectories during 10 min. A relatively low laser intensity was used to minimize laser-induced cellular damage. All acquisitions were performed using a temperature control system (“cube & box,” life imaging services, Basel, Switzerland) at 37 °C. VGlut1 time-lapse acquisitions were performed with a Leica Spinning disk with a ×40 oil immersion objective in a sequential mode with a time interval of 600 ms between each image during 5 min.

### Image analysis

#### Analysis of proteins containing vesicle motility

tPA and VAMP2 motility analysis was analyzed in both axonal and dendritic processes. Axons were distinguished from dendrites firstly based on known morphological characteristics: greater length, thin and uniform diameter, and sparse branching [58]. Neurons showing signs of stress by phase-contrast microscopy (PCM) are not included in our study. After each live imaging session, a retrospective immunocytochemistry was performed with an anti-MAP2 antibody to confirm the nature of each observed process. We selected the proximal to intermediate regions of axons and dendrites for time-lapse imaging analysis. Only those that appeared to be single axons and separate from other processes in the field were chosen for recording axonal transport. Regions where crossing or fasciculation occurred were excluded from analysis. A vesicle has been considered stationary if it remained immotile for the entire recording period; a vesicle has been considered mobile only if the displacement was ≥2 µm during 5 min.

#### Protein trafficking

tPA and VAMP2 trajectories were generated and analyzed using the KymoToolbox, an ImageJ plug-in developed by Fabrice P. Cordelières. This plug-in allows the generation of multiple kymographs that are calibrated in space (x axis in μm) and time (y axis in s). All of kymographs are generated here with a length of 60 µm during 10 min. Kymographs were then analyzed manually by drawing the vesicles trajectories. The plug-in extracts the dynamic parameters directly from the trajectories and also reports the tracked particles on the original video with a color directionality code (red for anterograde, green for retrograde and blue for stationary dynamics).

#### Protein fusion

tPA and VAMP2 fusion event were measured on kymographs after generation using KymoToolbox plug-in. Kymographs were generated with various length but like protein trafficking only during 10 min. VGlut1 fusion were measured using detect particles from the ComDet plug-in on FIJI. ComDet quantified each picture from each time-lapse acquisition, and finally a mean of VGlut1-SEP positives puncta was made for each process.

### Experimental design and statistical analyses

The values of charts are presented as mean ± SD or SEM. All analyses were completed from a minimum of two to nine independent cultures. Each experiment was performed from independent dish of cultured cortical neurons. The number of samples, named “n” corresponds to either the number of independent neurons analyzed (all figures excepted Fig. 3) or to the number of independent vesicles analyzed. Data have been analyzed with Prism (Graphpad) software. Shapiro-Wilk tests were used to ensure a normal distribution. Comparisons of two data sets were performed using unpaired two-tailed Student’s t-test for normally distributed data sets and Mann–Whitney test for non-normally distributed data sets. Comparisons for multiple data sets were performed using one-way analysis of variance with Tukey’s post hoc test for normally distributed data sets and Kruskal–Wallis test with Dunn’s multiple comparison test for non-normally distributed data sets. Significance levels were defined as *p < 0.05; **p < 0.01; ***p < 0.001; ****p < 0.0001, ns: not significant.

## Supplementary information

Checklist

Supplementary Legends

Figure S1

Figure S2

Figure S3

Figure S4

Figure S5

## Data Availability

All the data presented in this paper including raw data will be available by contacting the corresponding author, Pr Denis VIVIEN.
