# Supplementary material for: Modulations of the neuronal trafficking of tissue-type plasminogen activator (tPA) influences glutamate release
Source: Cell Death Dis. 2023 Jan 18;14(1):34. doi: 10.1038/s41419-022-05543-9 (PMC9845363; doi:10.1038/s41419-022-05543-9)
Supplement: Supplementary file 2 — Supplementary Legends [file 41419_2022_5543_MOESM2_ESM.docx]

**Legends of Extended Data figures**

**Extended Data Fig. 1: Neuronal activation decreases the vesicles distance travelled by increasing the time they spend on pause.**

**A**. Schematic representation and equation of the kymograph persistence indice. **B**, **E**. Persistence index of tPA containing vesicles in dendrites (**B**) and in axons (**E**) in basal conditions or neuronal activation (mean ± SEM). **C**, **F**. Minimal distance between the start and the end of each trajectory of dendritic (**C**) and axonal (**F**) tPA-containing vesicles (mean ± SD). **D**, **G**. Time spent by dendritic (**D**) and axonal (**G**) vesicles in the retrograde and the anterograde direction or in pause as percentage (mean ± SD). **B**,**C**,**D** Control n= 207 ; Bic/Gly n= 193; KCl n=157 vesicles from 10 to 13 neurons from 2 to 5 independent cultures. **E**,**F**,**G** Control n= 259; Bic/Gly n= 475; KCl n=451 vesciles from 11 to 20 neurons from 2 to 5 independent cultures. **B**-**G** Kruskal-Wallis’s test followed by a Dunn’s multiple comparisons test were used. ** p<0.05, ** p<0.01*** p*<0.005, ***** p*<0.0001, ns: not significant.

**Extended Data Fig. 2: Neuronal inhibition does not influence dendritic tPA-containing vesicles trafficking but increases the time spent by axonal tPA vesicles in the retrograde direction.**

**A**, **D**. Persistence index of tPA containing vesicles in dendrites (**A**) and in axons (**D**) in basal condition or neuronal inhibition (Error bars = SD). **B**, **E**. Minimal distance between the start and the end of each trajectory of dendritic (**B**) and axonal (**E**) tPA-containing vesicles (Error bars = SEM). **C**, **F**. Time spent by dendritic (**C**) and axonal (**F**) vesicles in the retrograde and the anterograde direction or in pause as percentage (Error bars = SD). **A**,**B**,**C** Control n= 201 ; DMSO n= 198; TTX n= 235 and CNQX/APV n= 182 vesicles from 11 to 14 neurons from 2 to 5 independent cultures. **D**,**E**,**F** Control n= 258 ; DMSO n= 690; TTX n= 463 and CNQX/APV n= 286 vesicles from 10 to 23 neurons from 2 to 5 independent cultures. **A**-**F** Kruskal-Wallis’s test followed by a Dunn’s multiple comparisons test were used. ** p<0.05, ** p<0.01*** p*<0.005, ***** p*<0.0001, ns: not significant.

**Extended Data Fig. 3: Only neuronal activation modifies endocytic and exocytotic events of tPA-containing vesicles.**

**A**. Representative kymographs of tPA-SEP traces in dendrites during chemical activation with following a schematic representation of analyses made on it. **B**, **C**. Number of tPA-SEP exocytosis (B) and endocytosis (C) events observed in dendrites during neuronal activation. **D**. Percentage of dendritic motile tPA-SEP positive vesicles. **E**. Pie charts representing the percentage of exocytosed HaloTag-tPA-SEP vesicles (SEP positives, in yellow) and intracellular HaloTag-tPA-SEP vesicles (SEP negatives, in magenta) in dendrites, (**B**-**E**) Control n=22, Bic/Gly n= 21, KCl n=16 neurons from 2 to 5 independent cultures. **F**. Representative kymographs of tPA-SEP traces in axons during chemical activation. **G**, **H**. Number of tPA-SEP exocytosis (G) and endocytosis (H) events observed in axons during neuronal activation. **I** . Percentage of axonal motile tPA-SEP positive vesicles. **J**. Pie charts representing the percentage of exocytosed HaloTag-tPA-SEP vesicles (SEP positive, in yellow) and intracellular HaloTag-tPA-SEP vesicles (SEP negative, in magenta) in axons, (**G**-**J**) Control n=12, Bic/Gly n= 14, KCl n=14 neurons from 2 to 5 independent cultures. **K**. Representative kymographs of tPA-SEP traces in dendrites during neuronal inhibition. **L**, **M**. Number of tPA-SEP exocytosis (L) and endocytosis (M) events observed in dendrites during neuronal inhibition. **N**. Percentage of dendritic motile tPA-SEP positives vesicles. **O**. Pie charts representing the percentage of dendritic exocytosed HaloTag-tPA-SEP vesicles (SEP positive, in yellow) and intracellular HaloTag-tPA-SEP vesicles (SEP negative, in magenta) in dendrites, (**L**-**O**) Control n=24, DMSO n=16, TTX n=27, CNQX/APV n=19 neurons from 2 to 6 independent cultures. **P**. Representative kymographs of tPA-SEP traces in axons during neuronal inhibition. **Q**, **R**. Number of tPA-SEP exocytosis (L) and endocytosis (M) events observed in axons during neuronal inhibition. **S**. Percentage of axonal motile tPA-SEP positive vesicles. **T**. Pie charts representing the percentage of exocytosed HaloTag-tPA-SEP vesicles (SEP positive, in yellow) and intracellular HaloTag-tPA-SEP vesicles (SEP negative, in magenta) in axons, (**Q**-**T**) Control n=13, DMSO n=17, TTX n=14, CNQX/APV n=16 neurons from 2 to 4 independent cultures. **B**-**D**, **G**-**I**, **L**-**N** and **Q**-**S**. Kruskal-Wallis’s test followed by a Dunn’s multiple comparisons test were used. **A**, **F**, **K** and **P** kymograph scale: x=40µm, y=10min. ** p<0.05, ** p<0.01*** p*<0.005, ***** p*<0.0001, ns: not significant.

**Extended Data Fig. 4: Neuronal activation decreases the vesicles distance travelled by increasing the time they spend on pause.**

**A**, **B**. Time spent by dendritic (A) and axonal (B) vesicles in the retrograde and the anterograde direction or in pause as percentage in basal conditions or neuronal activation (Error bars = SD). A. Control tPA n= 106; Control VAMP2 n= 106; Bic/Gly tPA n= 154; Bic/Gly VAMP2 n= 155 vesicles for retrograde trafficking and Control tPA n= 177; Control VAMP2 n= 112; Bic/Gly tPA n= 166; Bic/Gly VAMP2 n= 144 vesicles for anterograde trafficking from 10 to 15 neurons from 3 to 5 independent cultures. B. Control tPA n= 200; Control VAMP2 n= 108; Bic/Gly tPA n= 379; Bic/Gly VAMP2 n= 70 vesicles for retrograde trafficking and Control tPA n= 104; Control VAMP2 n= 168; Bic/Gly tPA n= 185; Bic/Gly VAMP2 n= 136 vesicles for anterograde trafficking from 10 to 15 neurons from 3 to 6 independent cultures. **C**, **D**. Time spent by dendritic (C) and axonal (D) vesicles in the retrograde and the anterograde direction or in pause as percentage in basal condition or neuronal inhibition (Error bars = SD). C. Control tPA n= 181; Control VAMP2 n= 110; CNQX/APV tPA n= 155; CNQX/APV VAMP2 n=118 vesicles for retrograde trafficking and Control tPA n= 180; Control VAMP2 n= 119; CNQX/APV tPA n= 169; CNQX/APV VAMP2 n= 113 vesicles for anterograde trafficking from 11 to 13 neurons from 2 to 5 independent cultures. D. Control tPA n= 549; Control VAMP2 n= 67; CNQX/APV tPA n= 252; CNQX/APV VAMP2 n=120 vesicles for retrograde trafficking and Control tPA n= 250; Control VAMP2 n= 140; CNQX/APV tPA n= 81; CNQX/APV VAMP2 n= 219 vesicles for anterograde trafficking from 10 to 17 neurons from 3 to 5 independent cultures. **A**-**D** Kruskal-Wallis’s test followed by a Dunn’s multiple comparisons test were used. ** p<0.05, ** p<0.01*** p*<0.005, ***** p*<0.0001, ns: not significant.

**Extended Data Fig. 5: Dynamic axonal vesicles spend more time off when neurons display tauopathy.**

**A**, **F**. Representative kymographs of dendritic (**A**) and axonal (**F**) tPA containing vesicles in WT neurons and Tau22 neurons **B**, **F**. Persistence index of tPA containing vesicles in dendrites (**B**) and in axons (**F**) in WT or Tau22 neurons (Error bars = SEM). **C**, **G**. Minimal distance between the start and the end of each trajectory of dendritic (**C**) and axonal (**G**) tPA containing vesicles (Error bars = SD). **D**, **H**. Time spent by dendritic (**D**) and axonal (**H**) vesicles in the retrograde and the anterograde direction or in pause as percentage (Error bars = SD). **B**, **C, D** WT n= 233; Tau22 n= 279 vesicles from 15 to 16 neurons from 3 to 4 independent cultures. **F**, **G, H** WT n= 342; Tau22 n= 453 vesicles from 13 to 14 neurons from 3 to 4 independent cultures. **B**, **C**, **D**, **F**, **G** and **H**. Samples were drawn from at least three independent experiments. Statistical tests: two-tailed Mann-Whitney test** p<0.05*, ***** p*<0.0001, ns: not significant.
