# Supplementary figures and images for: Modulations of the neuronal trafficking of tissue-type plasminogen activator (tPA) influences glutamate release
Source: Cell Death Dis. 2023 Jan 18;14(1):34. doi: 10.1038/s41419-022-05543-9 (PMC9845363; doi:10.1038/s41419-022-05543-9)

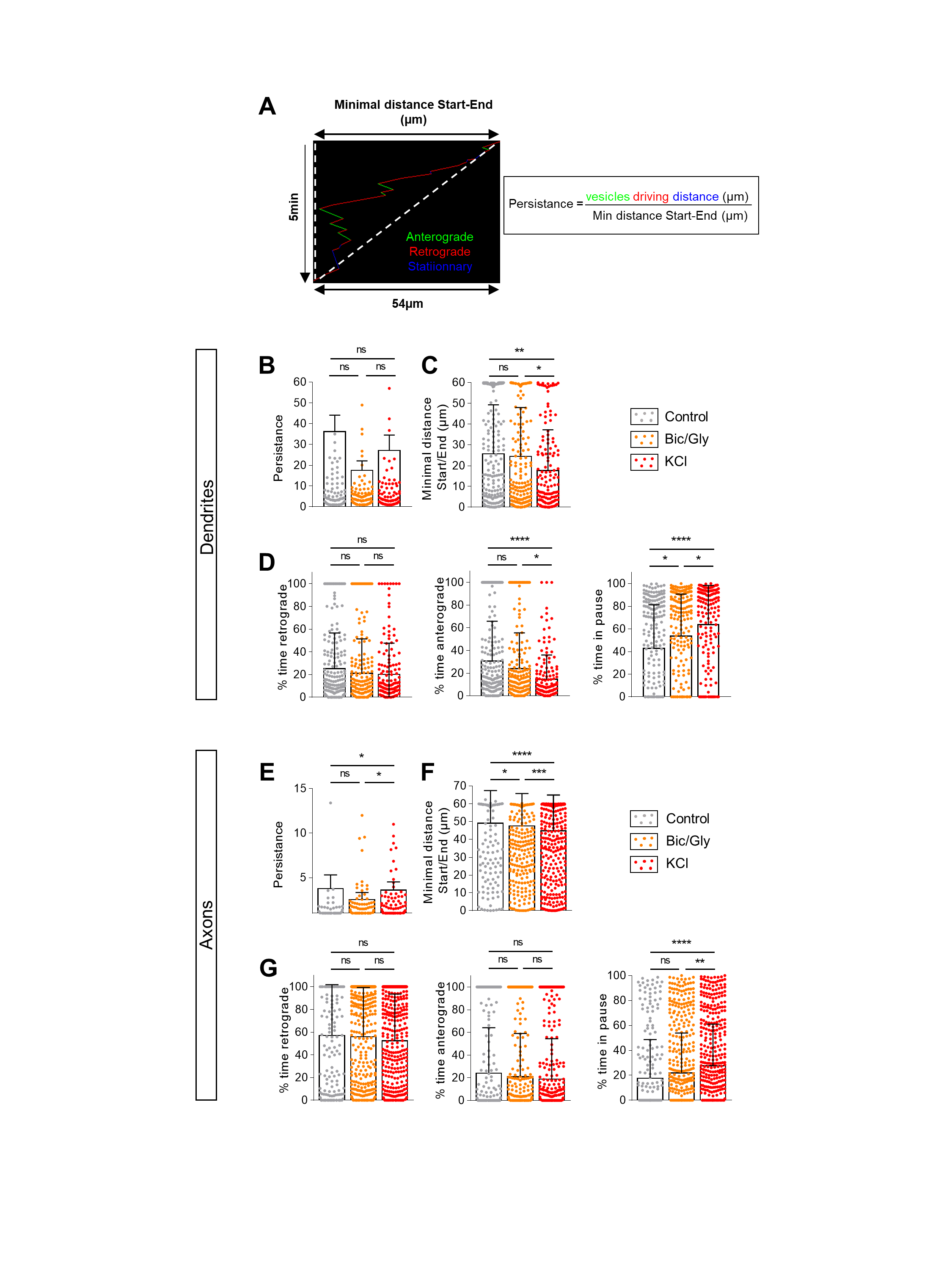

Supplement: Supplementary file 3 — Figure S1 [file 41419_2022_5543_MOESM3_ESM.tif]

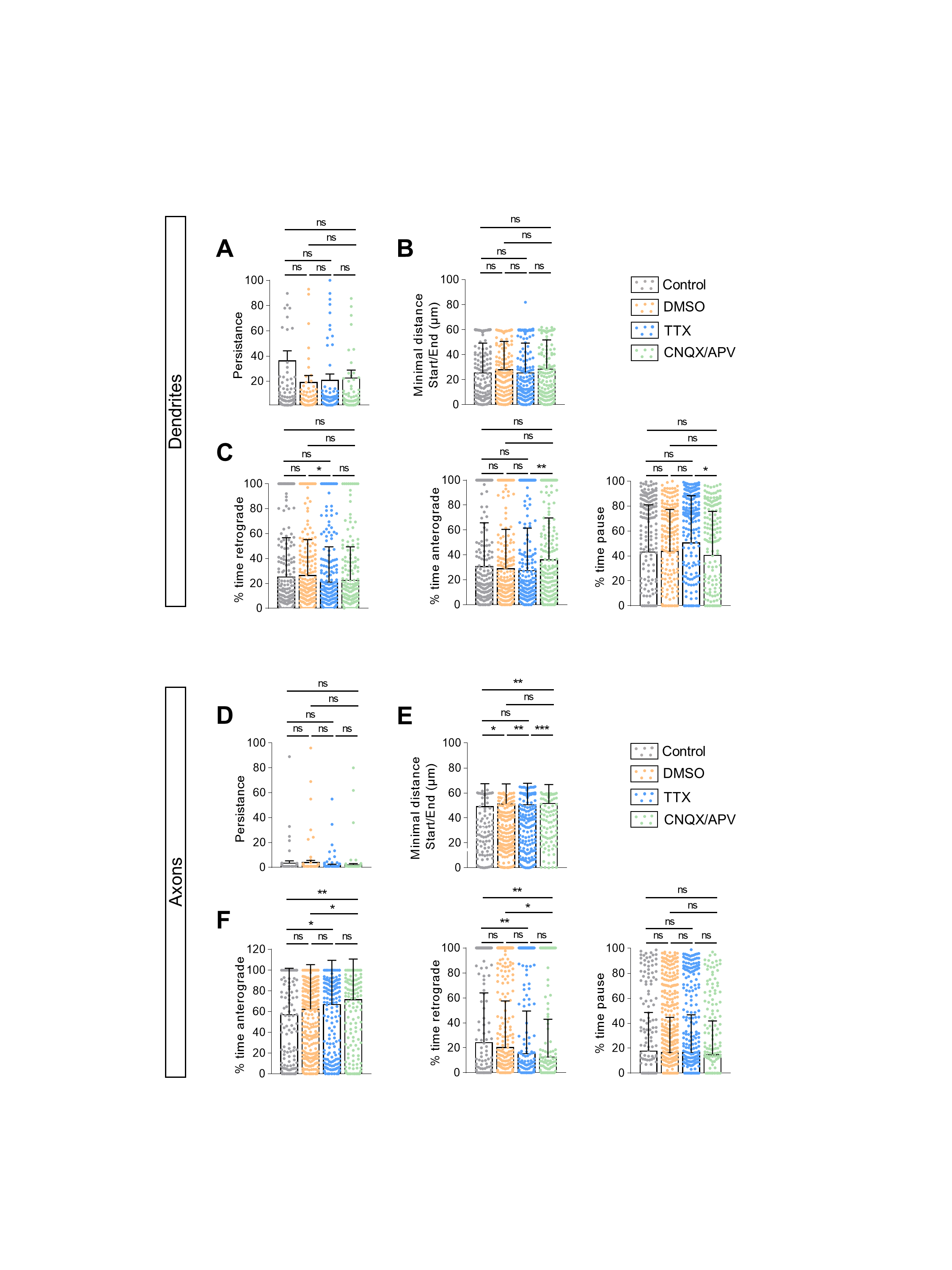

Supplement: Supplementary file 4 — Figure S2 [file 41419_2022_5543_MOESM4_ESM.tif]

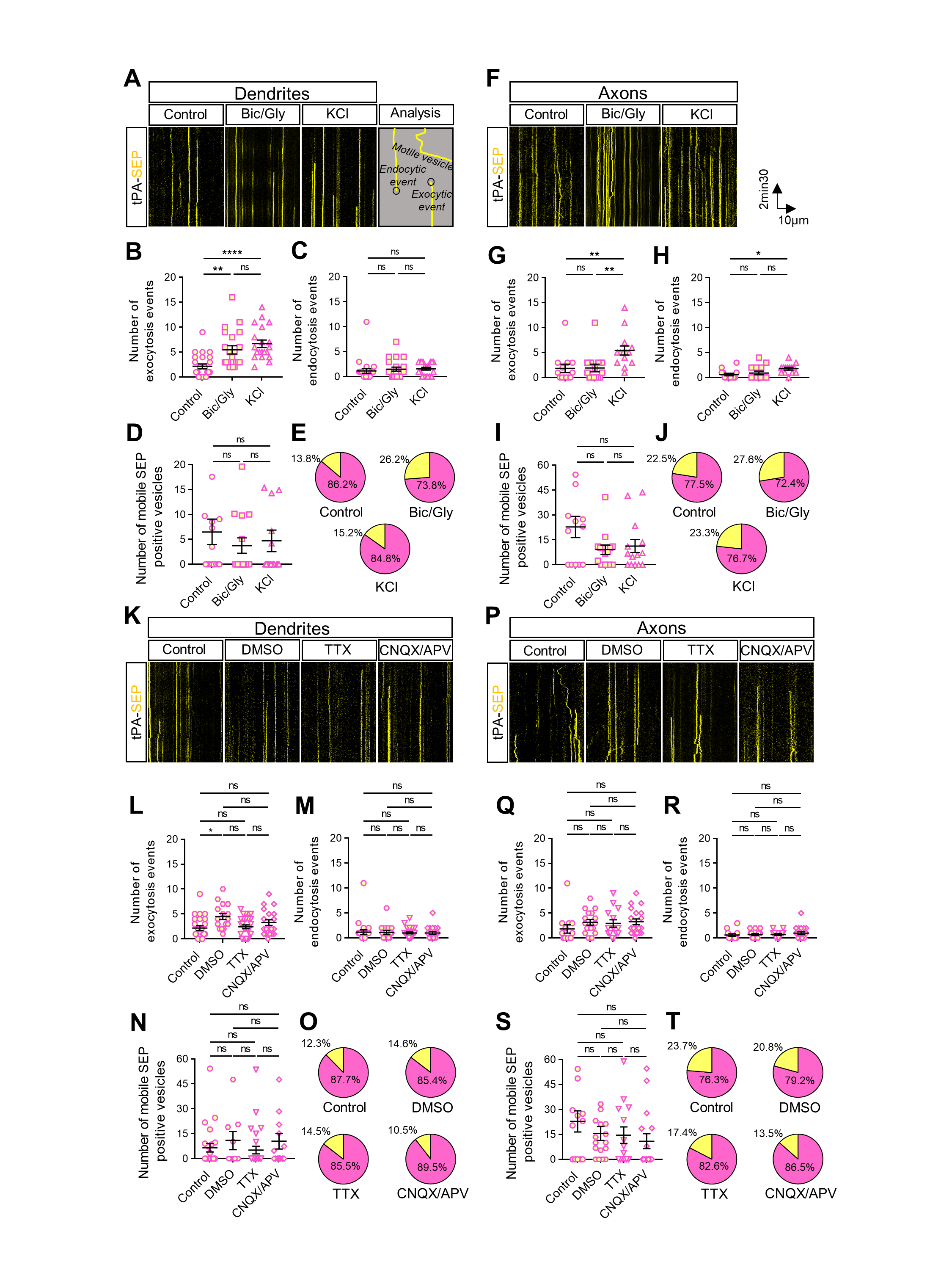

Supplement: Supplementary file 5 — Figure S3 [file 41419_2022_5543_MOESM5_ESM.tif]

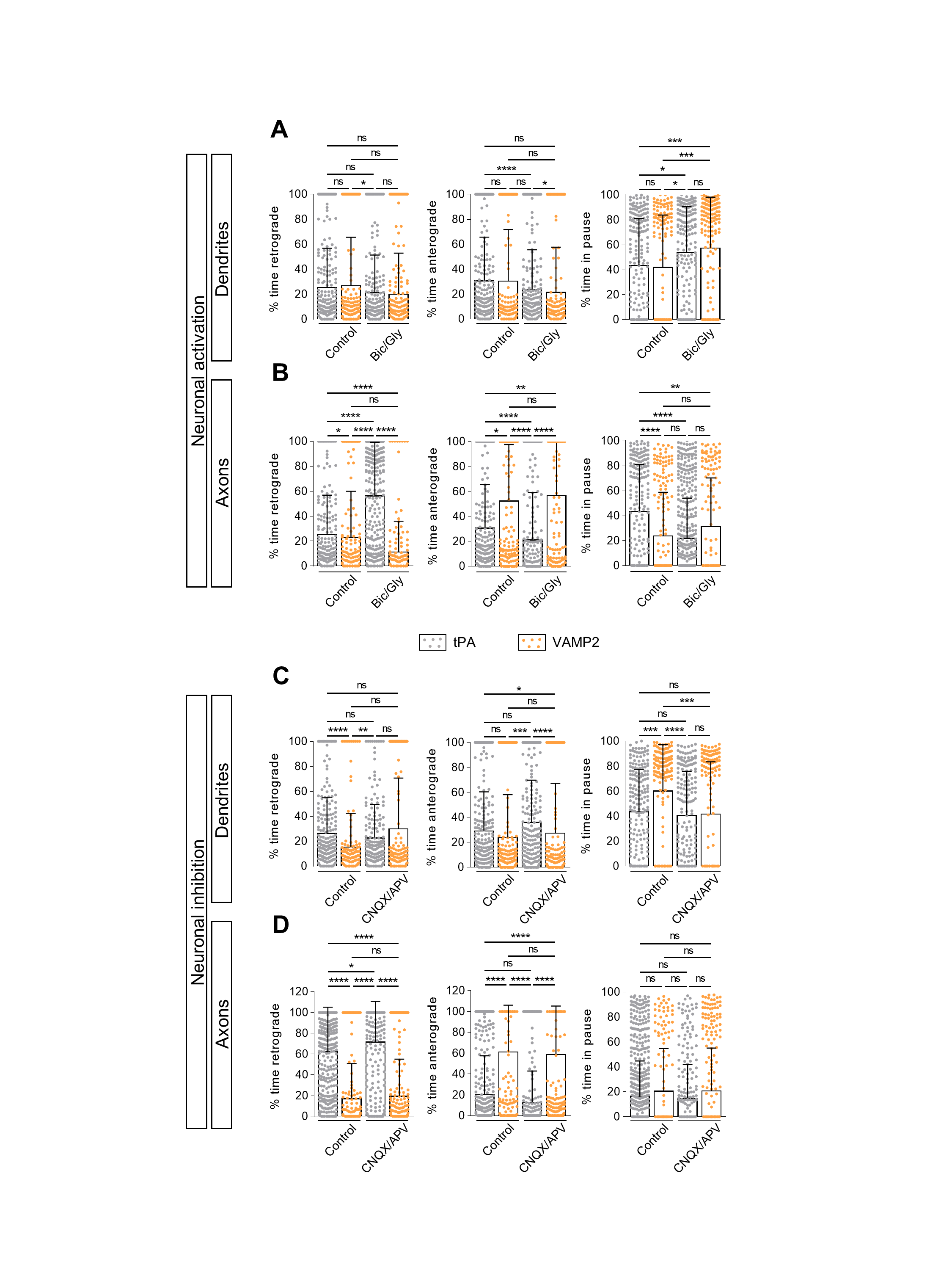

Supplement: Supplementary file 6 — Figure S4 [file 41419_2022_5543_MOESM6_ESM.tif]

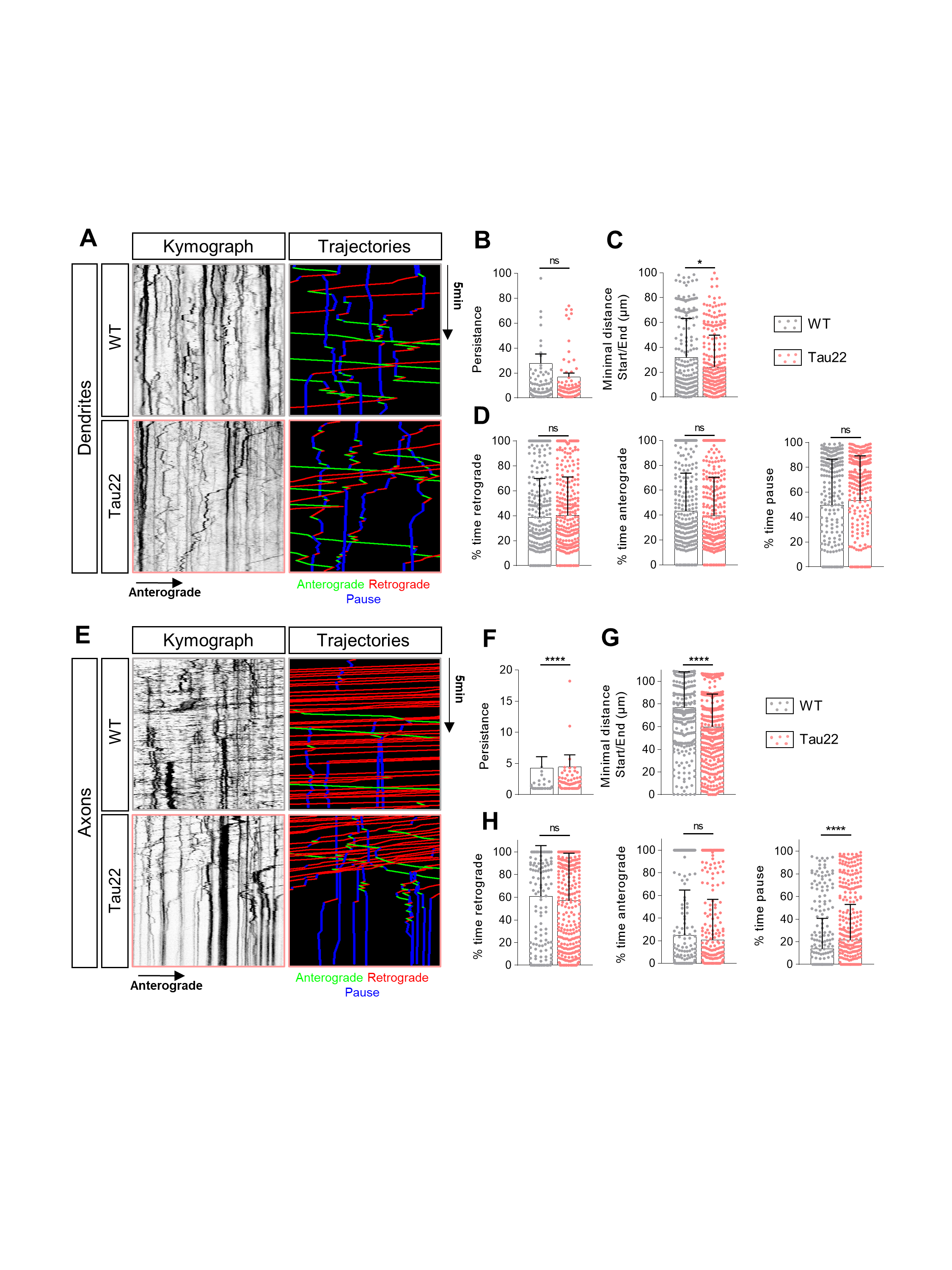

Supplement: Supplementary file 7 — Figure S5 [file 41419_2022_5543_MOESM7_ESM.tif]
